# A parallel algorithm for motion estimation in video coding using the bilinear transformation

**DOI:** 10.1186/s40064-015-1038-z

**Published:** 2015-06-24

**Authors:** Charalampos Konstantopoulos

**Affiliations:** Department of Informatics, University of Piraeus, 80 Karaoli and Dimitriou, Piraeus, Greece

**Keywords:** Motion estimation, Video coding, Parallel algorithms, Hypercube network

## Abstract

Accurate motion estimation between frames is important for drastically reducing data redundancy in video coding. However, advanced motion estimation methods are computationally intensive and their execution in real time usually requires a parallel implementation. In this paper, we investigate the parallel implementation of such a motion estimation technique. Specifically, we present a parallel algorithm for motion estimation based on the bilinear transformation on the well-known parallel model of the hypercube network and formally prove the time and the space complexity of the proposed algorithm. We also show that the parallel algorithm can also run on other hypercubic networks, such as butterfly, cube-connected-cycles, shuffle-exchange or de Bruijn network with only constant slowdown.

## Background

Motion estimation plays an important role in reducing the data redundancy typically existing between successive frames of a video and hence it is always included in any video compression scheme (Sayood 2012; Rao et al. 2014; Chiariglione 2012). It is also that step of compression algorithms with the highest computational demands.

The need for accurate estimation of the motion in a video is more pressing in compression techniques aiming at low or very low bit rates (Mokraoui et al. 2012; Ghanbari et al. 1995; Sayed and Badawy 2006). Inaccurate motion estimation increases the prediction error and thus more bits should be allocated for storing or transmitting this information. Thus, for this low-bit rate setting, simple block-matching motion estimation is not adequate due to its simplistic assumption about the motion of the objects in a video. Specifically, the basic assumption in this technique it that each video frame can be split into small square blocks. The motion at all the pixels of each block is the same, more precisely, purely translational and hence it can be described by only one vector per block. Clearly, this assumption is not realistic and as a result, simple block-matching motion estimation algorithms fail to identify the actual movement in a video especially when there is complex object movement in the scene.

In order to achieve more accurate motion estimation without overly increasing computational demands, a number of techniques have been proposed, which generalize the block-based algorithms (Mokraoui et al. 2012; Tekalp 1995; Aizawa and Huang 1995; Altunbasak and Tekalp 1997; Huang et al. 2013; Kordasiewicz et al. 2007; Sharaf and Marvasti 1999; Nosratinia 2001; Sayed and Badawy 2004; Nakaya and Harashima 1994; Muhit et al. 2012). These techniques assume a regular tiling over the image where each tile can be triangular or rectangular. The movement of each tile is rendered more realistically than in simple block-based algorithms by employing more complex spatial transformations such as the affine, perspective or bilinear transformation (Wolberg 1990) or by employing elastic motion models (Muhit et al. 2010, 2012) which include the simple translation as a special case.

In a previous work (Konstantopoulos et al. 2000), we have designed a parallel algorithm on the parallel model of the hypercube network for motion estimation in video using the affine transformation. We have demonstrated how to perform this estimation with low time complexity as well as with low local memory requirements per processor. In this paper, we follow the general methodology in (Konstantopoulos et al. 2000), and present a parallel motion estimation algorithm based on the bilinear transformation again on the hypercube. Note that the bilinear transformation is more complex than the affine one since the latter is a special case of the former. Although, achieving low time and space complexity again is more difficult now due to the increased complexity of the bilinear transformation, we will formally prove that the proposed parallel implementation achieves similar low complexity as in the case of the affine transformation.

The rest of the paper is organized as follows. In “Related work”, relevant work is presented. In “Spatial transformations and motion estimation”, motion estimation based on the bilinear transformation is discussed while in “The parallel algorithm”, the parallel algorithm for this motion estimation is presented and its time and space complexity is analyzed. Finally, “Conclusions” concludes our work.

## Related work

Video coding is the enabling technology for nearly all multimedia applications (Sayood 2012). Acknowledging this fact, a number of standardization efforts have taken place during the last 25 years, which constantly improve the rate-distortion trade-off in the lossy compression applied in video coding (Rao et al. 2014). The core technique for reducing data redundancy in video is the motion compensated prediction where the contents of each frame are predicted from the contents of one or two reference frames, taking also into account the movements of the objects between these frames. Thus, accurately estimating the motion in a scene reduces the prediction error, helps in reducing the data redundancy and hence achieves higher compression ratios. Considering the complexity of this estimation, most video coding standards follow a compromise solution by dividing each frame into a number of blocks, termed macroblocks, and then assume a simple translational motion where the motion of each macroblock can be expressed by a single motion vector. As has been mentioned in “Background”, a large body of literature have appeared, which propose improved motion estimation techniques by employing more advanced motion models, however, with increased computational complexity.

Due to heavy computation demands of video coding, parallel implementation of the basic operations of this computation is necessary for satisfying the real time constraints usually imposed in multimedia applications. Fortunately, motion estimation within each macroblock, which is the most computation intensive task in video coding, exhibits data parallelism, that is, different data can be processed concurrently by multiple processors. Nevertheless, the use of previous frames or previous macroblocks in the same frame for encoding the current frame or macroblock, respectively makes video coding an inherently sequential procedure at a higher level, limiting the degree of parallelism that can be achieved. Yet, for limiting the effect of data loss in a frame due to transmission errors in all subsequent frames, or for providing random access capability in the encoded video, most video coding standards define segments within video that can be processed independently, that is, they do not depend on previously decoded parts of the video. Specifically, the frame sequence can be spit into a number of group of pictures (GOPs), each of which contains consecutive frames which can be encoded/decoded independently of other groups. In addition, each frame can be divided into a number of slices each containing a number of consecutive macroblocks of the frame. Again, each slice can be encoded/decoded independently of other slices. Although, the aim for these partitioning techniques was not to facilitate parallel processing, the fact that GOPs and slices can be processed independently can also be exploited for effective parallel implementation. Also, in contrast to the previous video coding standards where parallel processing was only an afterthought, in the latest standard, HEVC (Sullivan et al. 2012), parallel processing is considered in the first place and additional partitioning schemes (tiling) or pipeline-based techniques (wavefront processing) are introduced (Pourazad et al. 2012). In tiling, each frame is partitioned into rectangular regions (tiles) separated by vertical and horizontal boundaries. Each tile can be processed independently of other tiles thereby enabling parallel processing. In wavefront processing, the processing of the current frame proceeds in raster scan order but the processing of a block in a row can start as soon as two neighboring blocks in the row above have been processed.

Parallel implementations for video encoders/decoders can be found either in hardware or software. In the first approach, application specific integration circuits (ASICs) are designed which implement specific functionalities in video coding (Malvar et al. 2003; Chen et al. 2006; Ruiz and Michell 2011; Badawy and Bayoumi 2002a, b). For instance, a large number of architectures have appeared for block matching motion estimation algorithms especially for the full search algorithm (Ruiz and Michell 2011; Ou et al. 2005; Bojnordi et al. 2006; Zhang and Gao 2007; Li et al. 2007; Lin et al. 2008; Kim and Park 2009; Chatterjee and Chakrabarti 2011). Due to its highly regular data flow, most implementations of this algorithm use mesh-like systolic arrays. Also, hardware architectures have been proposed in the literature for more accurate motion estimation using the affine transformation (Sayed and Badawy 2006; Badawy and Bayoumi 2002a, b; Utgikar et al. 2003). The main benefit of the hardware-based coders is the real-time performance. However, their shortcoming is the lack of flexility in case that some parameters of the computation need to change. In addition, they can easily become obsolete rather soon due to the rapid advances in video coding techniques.

The second implementation approach for video coding is the software implementation in general-purpose computing platforms (Fernandez and Malumbres 2002; Jung and Jeon 2008; Ahmad et al. 2001; Alvanos et al. 2011; Hsiao and Wu 2013) with particular focus on GPU implementations (Cheung et al. 2010; Ren et al. 2010; Chen and Hang 2008; Kung et al. 2008; Pieters et al. 2009; Su et al. 2014). Although, a hardware based solution is always superior in computation speed, the ever-increasing number of cores in modern processors enables a cost-effective implementation of the basic functionalities of video coding with performance comparable to that of hardware coders/decoders.

In this paper, we deal with the problem of motion estimation in video by using the bilinear spatial transformation. Specifically, we propose a parallel algorithm for this computation on the well-known parallel model of the hypercube network (Leighton 1992). This network as well as its numerous variations have been intensively studied in the literature (Hsieh and Lee 2010; Shih et al. 2008; Fu 2008; Lai 2012; Kuo et al. 2013; Zhou et al. 2015). The rich interconnection structure of this network favours the design of “elegant” parallel algorithms for a number of problems (Grama et al. 2002) which can be used in other parallel models (Sundar et al. 2013), as well. Following the basic methodology of (Konstantopoulos et al. 2000), we present a hypercube algorithm with low communication and computation cost. We formally prove those good features and we also analytically determine the memory required per processor for running the algorithm.

## Spatial transformations and motion estimation

The motion estimation techniques employed in video coding split each frame into small regions, usually polygons, and then they estimate a number of motion parameters for each region. Next, the current frame $I_{n}$ is predicted from the previous decoded frame $\tilde{I}_{n - 1}$ by applying image warping (also known as texture mapping) (Wolberg 1990). This step can be expressed as follows:

1 $${\bar{I}}_{n} (x,y) = {\tilde{I}}_{n - 1} \left(f(x,y),\;g(x,y)\right)$$

where $\bar{I}_{n}$ is the prediction for the current frame and $x^{\prime} = f({x,y})$, $y^{\prime} = g({x,y})$ are the transformation functions which describe the on-going movement.

For instance, in the case of block matching algorithms, the functions $f$ and $g$ are given by the following relations:

2 $$\begin{aligned}f({x,y}) = x - u_{i} \hfill \\ g({x,y}) = y - v_{i}\end{aligned}$$

where $({u_{i},v_{i}})$ is the displacement vector for the $i$-st region (block).

When, coordinates $x^{\prime}$ and $y^{\prime}$ are not integers, the intensity value $\tilde{I}_{n - 1} ({x^{\prime},y^{\prime}})$ is derived by applying an interpolating function on the intensities of the nearest image pixels. In this function, the intensity value for the point $({x^{\prime},y^{\prime}})$ is given by the following relation:

3 $$\begin{array}{*{20}l} {\tilde{I}_{n - 1} (x^{\prime},y^{\prime}) = (1 - a)((1 - b)\tilde{I}_{n - 1} (\lfloor {x^{\prime}} \rfloor,\lfloor {y^{\prime}} \rfloor) +} \\ {b\tilde{I}_{n - 1} (\lfloor {x^{\prime}} \rfloor,\lfloor {y^{\prime}} \rfloor + 1)) +} \\ {a((1 - b)\tilde{I}_{n - 1} (\lfloor {x^{\prime}}\rfloor + 1,\lfloor {y^{\prime}}\rfloor) +} \\ {b\tilde{I}_{n - 1} (\lfloor {x^{\prime}}\rfloor + 1,\lfloor {y^{\prime}} \rfloor + 1))} \\ \end{array}$$

where $a$ and $b$ are the fractional part of the coordinates $x^{\prime}$ and $y^{\prime}$, respectively.

Different motion estimation methods can be developed according to the spatial transformation assumed in the estimation. Clearly, the employed transformation largely determines the accuracy of the motion estimation. Besides the estimation accuracy, the spatial transformation should be formulated with a relevant small number of parameters so that its estimation does not require a lot of numerical operations. However, these are conflicting objectives since high accuracy in motion estimation usually demand more complex transformation functions. A clear benefit of the parallel motion estimation is that more complex options can be adopted while keeping the execution time reasonably low.

In general, the texture mapping operation comprises the following steps (Nakaya and Harashima 1994; Huang and Hsu 1994):

1. Estimation of motion parameters for each region of the frame.
2. Estimation of the value of the transformation functions at all frame pixels based on the above parameters.
3. Interpolation for finding the intensity of the image in the frame $\tilde{I}_{n - 1}$ of these pixels that were not mapped to integer coordinates after applying the spatial transformation.

The estimation of motion parameters usually requires an iteration of the second and third step in order that the optimal values for the motion parameters can be determined. It is now clear that texture mapping is rather a costly operation. Fortunately, this kind of operation is amenable to massive parallelism since computations at different pixels can be executed in parallel most of time.

### Bilinear transformation

**Figure 1:**
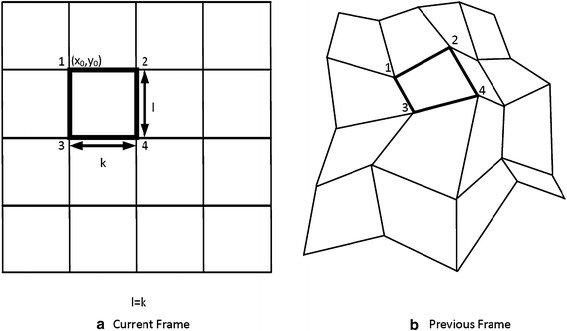
Motion estimation between the current frame (**a**) and its previous frame (**b**) based on the bilinear transformation.

Although, many different spatial transformation have been studied in Graphics, three transformations have been commonly used (Tekalp 1995; Sharaf and Marvasti 1999), for video compression, namely, the affine, the bilinear and the perspective transformation. In this work, we will focus on the bilinear transformation. In this transformation, the mapping functions $f$ and $g$ are given as follows:

4 $$\begin{aligned}f(x,y) = a_{i1} x + a_{i2} y + a_{i3} xy + a_{i4} \hfill \\ g(x,y) = a_{i5} x + a_{i6} y + a_{i7} xy + a_{i8}\end{aligned}$$

where $a_{i1} \ldots a_{i8}$ are the eight parameters of this transformation. Clearly, if the values of $f$ and $g$ are given for four points of the image, the parameters $a_{i1} \ldots a_{i8}$ can be determined by solving two linear systems, each of four equations with four unknowns. For this reason, when using bilinear transformation, it is most convenient to split the image into rectangular regions (Figure 1a). Then, by giving the displacement vectors at the corners of each rectangle, the parameters of the bilinear transformation for that rectangle can be easily derived. Another reason for using rectangular regions is that the bilinear transformation maps the vertical and horizontal lines again to lines (Wolberg 1990). For all other orientations, this does not hold, and, for instance, a diagonal line is transformed to a curve. Another interesting property of that transform that can be easily verified is that the boundaries of the objects are preserved after this transformation, that is, the pixels on the border of each region are again on the border of the image of this region after the application of the transform. Finally, as already mentioned above, the affine transformation is a special case of the bilinear transformation by setting $a_{i3}$ and $a_{i7}$ equal to $0$.

Now, if $\vec{d}_{1} = ({d_{1}^{x},d_{1}^{y}})$, $\vec{d}_{2} = ({d_{2}^{x},d_{2}^{y}})$, $\vec{d}_{3} = ({d_{3}^{x},d_{3}^{y}})$, $\vec{d}_{4} = ({d_{4}^{x},d_{4}^{y}})$ are the displacement vectors of the four corners of the block whose upper left corner is at point $(x_{0},y_{0})$ (the thick block) in Figure 1a, then the parameters $a_{i1},a_{i2}, \ldots,a_{i8}$ of the bilinear transformation for these displacements will be:

5 $$\begin{aligned} a_{i1} &= \frac{{({d_{2}^{x} - d_{1}^{x}})({l - y_{0}}) + y_{0}({d_{4}^{x} - d_{3}^{x}}) + kl}}{kl} \\ a_{i2} &= \frac{{({x_{0} + k})({d_{1}^{x} - d_{3}^{x}}) + x_{0}({d_{4}^{x} - d_{2}^{x}})}}{kl} \\ a_{i3} &= \frac{{d_{2}^{x} - d_{1}^{x} + d_{3}^{x} - d_{4}^{x}}}{kl} \\ a_{i4} &= x_{0} + d_{1}^{x} - a_{i1} x_{0} - a_{i2} y_{0} - a_{i3} x_{0} y_{0} \hfill \\ a_{i5} &= \frac{{({l - y_{0}})({d_{2}^{y} - d_{1}^{y}}) + y_{0}({d_{4}^{y} - d_{3}^{y}})}}{kl} \\ a_{i6} &= \frac{{({x_{0} + k})({d_{1}^{y} - d_{3}^{y}}) + x_{0} ({d_{4}^{y} - d_{2}^{y}}) + kl}}{kl} \hfill \\ a_{i7}& = \frac{{d_{3}^{y} - d_{1}^{y} + d_{2}^{y} - d_{4}^{y}}}{kl} \\ a_{i8} &= y_{0} + d_{1}^{y} - a_{i5} x_{0} - a_{i6} y_{0} - a_{i7} x_{0} y_{0}\end{aligned}$$

Another important issue in this motion estimation approach is the assumption about the movements of the adjacent blocks. Specifically, there is the continuous and the discontinuous motion model (Nakaya and Harashima 1994; Huang and Hsu 1994). In the first model, there is a correlation between the movement of adjacent blocks while in the second model the blocks are moving independently. For the same number of blocks, the continuous model requires a smaller number of bits for coding the motion parameters than the discontinuous model since the assumption of motion continuity reduces the degrees of freedom of the problem at hand. For this reason, the continuous model is commonly used for motion estimation in low-bit rate video coding schemes. This is also the model, we assume in this work. Thus, after the application of the bilinear transformation, the blocks are not overlapping while the relevant positions of the corners of each block are maintained, for instance, the upper left corner cannot be found lower than the lower left corner or right of the upper right corner (Figure 1b).

Since, the displacement vectors cannot be arbitrary large due to short time interval between successive frames in a video, we will consider the following range of values for the displacement vectors:

6 $$\begin{aligned}- \frac{k}{2} < d_{1}^{x},d_{2}^{x},d_{3}^{x},d_{4}^{x} < \frac{k}{2}\hfill \\ - \frac{l}{2} < d_{1}^{y},d_{2}^{y},d_{3}^{y},d_{4}^{y} < \frac{l}{2}.\end{aligned}$$

Notice also that with these displacement vectors, all the constraints of the continuous model are respected.

Now, we can prove the following lemma:

#### **Lemma 3.1**

*Given the constraints* (*6*)*, it holds that*$\frac{1}{k} < a_{i1} + a_{i3} y < 2 - \frac{1}{k}$, $\frac{1}{l} < a_{i6} + a_{i7} x < 2 - \frac{1}{l}$, $- \frac{l - 1}{k} < a_{i5} + a_{i7} y < \frac{l - 1}{k}$*where*$y \in [y_{0} - l \ldots y_{0}]$*and*$x \in [x_{0} \ldots x_{0} + k]$.

#### *Proof*

We will prove only the first inequality. The proof for the second and the third inequality is similar. From the Eq. (5) we get that:

7 $$a_{i1} + a_{i3} y = \frac{{({d_{2}^{x} - d_{1}^{x}}){\kern 1pt} \,({l - y_{0} + y}) +({d_{4}^{x} - d_{3}^{x}})\,({y_{0} - y}) + kl}}{kl}$$

It can be easily seen that $({l - y_{0} + y})$, $({y_{0} - y})$ and $lk$ are all non negative. Therefore, the expression (7) gets its maximum (minimum) value when the expressions $({d_{2}^{x} - d_{1}^{x}})$ and $({d_{4}^{x} - d_{3}^{x}})$ get their maximum (minimum) value. Given the constraints (6) and since these vectors always have integer coordinates, the maximum value for the expressions $({d_{2}^{x} - d_{1}^{x}})$ and $({d_{4}^{x} - d_{3}^{x}})$ is $k - 1$ while its minimum is $- k + 1$. Now, it is easy to see that the minimum value of (7) is $\frac{1}{k}$ and its maximum value is $2 - \frac{1}{k}$.$\square$

Now, if the coordinates of the point $({f({x,y}),g({x,y})})$ are not integers, the intensity value at that point is derived by applying the interpolation function (3) on the adjacent pixels of the frame $\tilde{I}_{n - 1}$. 

Algorithm 1 provides the basic steps for the motion estimation using the bilinear transformation (Nakaya and Harashima 1994). Specifically, for each of the frame blocks, all feasible combinations of displacement vectors at its corners are considered while respecting the constraints (6). For each combination, the parameters of the bilinear transformation are estimated and then the texture mapping step is performed (see also Figure 1). The error of prediction of the current frame from the previous one after this mapping is calculated and finally, for each block, the displacement vectors yielding the lowest prediction error are returned. This set of vectors is exactly the information that will be given to the decoder for restoring the current frame from the previous one by simply reversing the texture mapping step.

In the following section, the parallel algorithm on the hypercube network model for the above motion estimation is presented and its time and space complexity is analytically determined. 

## The parallel algorithm

A hypercube of $N(= 2^{n})$ nodes is an interconnection network where each network node is directly connected to $n$ other nodes whose binary representation differ from that of this node only at a single bit (Leighton 1992). Specifically, node $i(= i_{n - 1} i_{n - 2} \ldots i_{1} i_{0})$ is connected to the nodes $i^{(j)} (= i_{n - 1} \ldots \overline{{i_{j}}} \ldots i_{0})$ for $j = 0 \ldots n - 1$. Due to its rich interconnection, the hypercube has low diameter ($n$) and high bisection width ($N/2$). These features as well as the symmetry existing in the structure of this network facilitate the design of parallel algorithms with low communication cost.

Now, we assume video frames of dimension $N \times N$ where $N = 2^{n}$. We also assume a hypercube network of $N^{2}$ nodes and initially, the current and the previous frame have been distributed to the node/processors of this network. Specifically, pixel $(i,j)$ has been stored in the processor $j + iN$. For convenience, we view the hypercube as a two dimensional $N \times N$ mesh and thus the processor $j + iN$ can be considered as the processor $(i,j)$ of this mesh ($i,j = 0 \ldots N - 1$). It can also be easily seen that the processors along the same row or column of the mesh form a sub-hypercube of $N$ nodes and thus, wherever in the text, we mention columns and rows, we will actually mean the corresponding sub-hypercubes.

With regard to the communication capabilities of the processors in the hypercube, we will consider two different possibilities. Specifically, we assume either that each processor sends or receives at most one packet at a time (*one*-*port capability*) or that each processor is able to send to or receive from all its port simultaneously (*all*-*port capability*). With all-port capability, similar communication operations executed in succession can be pipelined and this results in great reduction of the total communication time.

Now, our goal is to design an algorithm will low computational and the communication cost as well as with low memory requirements at each node. Besides giving the details of the algorithm, we will also formally prove the effectiveness of the algorithm with respect to the costs above.

As has already been explained in the previous section, the estimation of the parameters of the bilinear transformation for each block is an iterative procedure where at each iteration, a different combination of displacement vectors at the block corners is tested and then a texture mapping step from the current to the previous frame is executed until the vector combination with the minimum prediction error is found. Apparently, texture mapping is the most computationally intensive step and since it is executed repeatedly, its parallel implementation will largely speed up the whole computation. Thus, in this paper we mainly focus on the parallel implementation of this step.

Algorithm 2 gives the basic steps of the parallel texture mapping as a part of an iterative procedure where all possible combinations of displacement vectors are examined. Assuming that the feasible range of the displacement vectors has been previously broadcasted to all processors [$O(logN)$ time], all processors can now produce the different vector combinations in the same order and thus they can work on the same displacement vectors simultaneously. Thus, given the displacement vectors at a particular iteration, each processor can determine the corresponding parameters of the bilinear transformation of its block by (5). Then, for computing the prediction error $|I_{n} (x,y) - \tilde{I}_{n - 1} ({x^{\prime},y^{\prime}})|$, each processor $(x,y)$ needs to learn only the value $\tilde{I}_{n - 1} ({x^{\prime},y^{\prime}} )$, since the intensity $I_{n} (x,y)$ is already stored in the processor.

A straightforward approach for transferring this value to processor $(x,y)$ is for a processor “near” the point $(x^{\prime},y^{\prime})$ to send these data. Specifically, the processor $({\lfloor {x^{\prime}} \rfloor,\lfloor {y^{\prime}}\rfloor})$ could estimate the intensity value $\tilde{I}_{n - 1} (x^{\prime},y^{\prime})$ by getting the intensity of pixels stored in neighboring processors (if needed) and then it could send that intensity value to the processor $(x,y)$. The problem arising with this approach is that the processor $({\lfloor {x^{\prime}}\rfloor,\lfloor {y^{\prime}}\rfloor})$ should know the processors to which it should send the intensity value it has just estimated. Since, for each block, the parameters of the bilinear transformation are different, this processor should estimate the transform parameters of a number of different blocks in order that it can determine which pixels are mapped after truncation to its position. Moreover, even if only one block was mapped to the “area” of this processor and hence only one instance of bilinear transformation was to be applied, still, it would be possible that more than one pixels could be mapped on the same pixel due to the truncation of the transformation output to the nearest integer. This holds even without applying this truncation, since reversing the bilinear transformation requires the solution of a quadratic equation anyhow (Wolberg 1990).

In order to get around these difficulties, random access read (RAR) operation (Ranka and Sahni 2012) is used for performing the transfer above. This operation consists of two phases. At the first phase, each processor $(x,y)$ sends a packet containing its address to the processor $({\lfloor {x^{\prime}} \rfloor,\lfloor {y^{\prime}} \rfloor} )$. The processor $({\lfloor {x^{\prime}} \rfloor,\lfloor {y^{\prime}} \rfloor} )$ now knows where to send all the data required for calculating the intensity value $\tilde{I}_{n - 1} (x^{\prime},y^{\prime})$ and in the second phase, it sends these data to these processors.

In general, the RAR implementation requires a distributed sorting step where the packets to be sent are sorted according to the recipients’ addresses. All practical sorting algorithms on a $N$-node hypercube require $O(\mathop {log}\nolimits^{2} N)$ time and thus the total time complexity of a sort-based RAR operation is of the same order (Ranka and Sahni 2012). The main goal is to implement the RAR operation without resorting to a sorting operation by exploiting the properties of the bilinear transform. In the following section, we give more details of this implementation.

After receiving the pixels required for the computation of $\tilde{I}_{n - 1} (x^{\prime},y^{\prime})$, each processor $(x,y)$ computes the prediction error for its pixel. Then, these local errors are distributively added and the total prediction error for each block finally ends up at the processor located at the upper-left corner of the block. This transfer can be easily implemented with two rounds of parallel segmented prefix sum operations (Leighton 1992). Initially, the segmented prefix sum operations are performed along the columns of the frames with the segment length of each prefix-sum operation being the height of the blocks. Then, parallel segmented prefix-sum operations are carried out along the lines coinciding with the horizontal boundaries of the rectangles (see Figure 1a). The segment length of each “horizontal” prefix-sum operation is now the block width. Each segmented prefix-sum takes $O(logN)$ time at most and thus the total time for estimating the total prediction error is of the same order.

Then, each of the above upper-left processors updates the minimum prediction error if the current prediction error is the lowest seen so far. In this case also, they store the corresponding displacement vectors. Thus, after the end of all iterations, each of the upper-left processor will know the minimum prediction error for its block and which displacement vectors at the corners of the rectangle give the best prediction. 

### The RAR operation

Algorithm 3 gives the basic steps for the proposed implementation of the RAR operation. As has been mentioned previously, in the first phase, each processor $(x,y)$ sends a read request to the processor holding all the information required for calculating the intensity of the pixel $(x^{\prime},y^{\prime})$ in the previous frame $\tilde{I}_{n - 1}$ where $x^{\prime} = f(x,y)$ and $y^{\prime} = g(x,y)$ by (4). Since, $x^{\prime}$, $y^{\prime}$ may not be integers, they should be rounded to nearest integers and thus, the request is sent to the processor $(x_{int},\lfloor {y^{\prime\prime}}\rfloor)$ which is close to the position $(x^{\prime},y^{\prime})$ as will seen later. Also, by viewing the hypercube of $N^{2}$ nodes as a two dimensional mesh $N \times N$, routing of this request can be performed by using the well known technique of $X - Y$ routing. First, the $x$-coordinate is corrected and the packet is routed horizontally toward the destination column and then the packet is routed vertically to the final destination. After, the read request has arrived the processor $(x_{int},\lfloor {y^{\prime\prime}} \rfloor)$, the second phase starts and the processor $(x_{int},\lfloor {y^{\prime\prime}} \rfloor)$ gathers all the pixels needed for estimating the intensity $\tilde{I}_{n - 1} (x^{\prime},y^{\prime})$ for all processors $(x,y)$ which sent read-requests to that processor. Then, it sends these pixels back to the above processors $(x,y)$ by reversing the steps of the $X - Y$ routing of the first phase. In what follows, we give the details of these steps.

#### *X*-routing

At this step, each processor $(x,y)$ sends a packet containing its coordinates to the processor $({\lfloor {x^{\prime}}\rfloor,y})$ except possibly when the processor is near the left edge of its block. Specifically, for these processors, the pixel $({\lfloor {x^{\prime}} \rfloor,y} )$ may be outside the image of the block in the previous frame. Thus, these processors $(x,y)$ are forced to send to the processor $({\lceil {x^{\prime}} \rceil,y} )$. We should specially treat these processors in order to ensure that after the end of $X$-routing, each processor will have received packets originated only from a single block. As will be seen, with this guarantee, the implementation of $Y$-routing is greatly simplified. Notice also that each processor can easily identify this special case. For instance, a processor $(x,y)$ inside the thick block of Figure 1a should send the packet to the processor $({\lceil {x^{\prime}} \rceil,y})$, only if $\lfloor {x^{\prime}} \rfloor < a_{i1} x_{0} + a_{i2} y_{0} + a_{i3} x_{0} y_{0} + a_{i4}$. Now, we prove the following Lemma.

##### **Lemma 4.1**

*Let*$(x_{1},y)$*and*$(x_{2},y)$*be two processors along the same horizontal line and let processors*$(x_{1}^{int},y)$, $(x_{2}^{int},y)$*be the recipients of the packets of these processors respectively during*$X$-*routing where*$x_{i}^{int}$*is either*$\lfloor {x^{\prime}} \rfloor$*or*$\lceil {x^{\prime}} \rceil$*depending on whether the above special case arises or not. If*$x_{1} < x_{2}$, *then it holds that*$x_{1}^{int} \le \;x_{2}^{int}$.

##### *Proof*

We consider two cases: (a) the processors $(x_{1},y)$ and $(x_{2},y)$ belong to the same block $B_{i}$ and (b) belong to different blocks. Now, we deal with the first case. Writing the first of the relations (4) as follows:

8 $$x^{\prime} = ({a_{i1} + a_{i3} y})x + a_{i2} y + a_{i4}$$

We notice that the terms $a_{i1} + a_{i3} y$ και $a_{i2} y + a_{i4}$ are constant for all processors of $B_{i}$ residing on the same horizontal line. Due to Lemma 3.1, the expression $a_{i1} + a_{i3} y$ is positive, therefore, $x^{\prime}_{1} < \;x^{\prime}_{2}$ and thus $x_{1}^{int} \le \;x_{2}^{int}$.

For the second case where processors $(x_{1},y)$ and $(x_{2},y)$ belong to different blocks, notice that because of the assumption of the continuous motion model and also due to the guarantee that each packet from a block ends up again inside the image of the block, the destinations of packets originated from different blocks are ordered according to the relevant locations of the blocks they belong to. Specifically, the block of processor $(x_{1},y)$ is left of the block of processor $(x_{2},y)$ and thus the packet of the former will end up left of the packet coming from the latter. Therefore, we have proved the Lemma for the second case as well. $\square$

If the destinations of the packets to be routed on the hypercube are already sorted with respect to their destinations, as in our case, the packet routing can be performed optimally in $O(logN)$ time by using monotone routing (Leighton 1992). Here, we assume that the packet destinations are all different. Otherwise, if $L$ is the maximum number of packets that have the same destination, then monotone routing is completed in $O(LlogN)$ time ($O(L + logN)$) in case of the one (all) port capability where

9 $$L = \mathop { \hbox{max} }\limits_{{B_{i}}} \mathop { \hbox{max} }\limits_{{\begin{array}{*{20}c} {line\;y = y_{q}} \\ {crosses\;B_{i}} \\ \end{array}}} \hbox{max} \left({\left({\frac{1}{{a_{i3} y_{q} + a_{i1}}}}\right),1}\right)$$

However, all packets having the same final destination after $X - Y$ routing, originating also from processors on the same horizontal line can be easily combined into a single proxy packet. Indeed, the source processors of these packets are consecutive along the horizontal line and thus their packets can be combined in $O(logN)$ time using standard techniques described in (Leighton 1992; Ranka and Sahni 2012). Then, only the proxy packet needs to be routed by $X - Y$ routing. The time complexity is given by the above expressions again but now

10 $$L = \mathop { \hbox{max} }\limits_{{B_{i}}} \mathop { \hbox{max} }\limits_{{\begin{array}{*{20}c} {line\;y = y_{q}} \\ {crosses\;B_{i}} \\ \end{array}}} \frac{{ \hbox{min} (|a_{i7} y_{q} + a_{i5} |,1)}}{{ \hbox{min} (a_{i3} y_{q} + a_{i1},1)}}$$

By Lemma 3.1, the expression $a_{i3} y_{q} + a_{i1}$ is in the range $[\frac{1}{k},\,2 - \frac{1}{k}]$ and is getting closer to $\frac{1}{k}$ when the four corners of $B_{i}$ tend to be collinear along the same vertical line after the application of bilinear transformation. In contrast, the value of this expression is getting nearer $2 - \frac{1}{k}$, when the corners of $B_{i}$ are moving apart horizontally. For the expression $|a_{i7} y_{q} + a_{i5} |$, its value is always in the range $\left[0,{\kern 1pt} \,\frac{l - 1}{k}\right]$ again by Lemma 3.1. It converges toward zero when the upper and the lower edge of the block $B_{i}$ still remain horizontal after the bilinear transformation while it converges toward $\frac{l - 1}{k}$ when the upper and the lower edge of the block $B_{i}$ are inclined $45^{\circ}$ after applying the transform. Overall, the maximum value of $L$ is $(l - 1)$ and this value results when the four corners of a block all converge to the same vertical line. For other more “typical” cases of corner displacements, $L$ takes much lower values.

Also, it is clear that after the end of $X$-routing, each processor have received at most $L$ packets and thus it requires that much local memory.

We can also provide an implementation of the $X$-routing with lower communication cost but with higher computation cost. Specifically, we can combine into a single proxy packet, all the packets coming from processors $(x,y)$ having the same destination $(x_{int},y)$. Thus, the communication time required for $X$-routing is now lower, namely, $O(logN)$. All these processors are consecutive along the same horizontal line and the proxy packet needs to carry only the interval $[x_{r} \ldots x_{q}]$ of these processors whose length is obviously $O(L)$ where $L$ is given by (9). In addition, all these processors belong to the same block of the frame $I_{n}$ and thus the processor $(x_{int},y)$ can easily identify that block from the above interval of $x$-values. Thus, then it is able to estimate the parameters $a_{i1}, \ldots,a_{i8}$ of the bilinear transformation for that block. Next, processor $(x_{int},y)$ can determine all the subintervals of the $[x_{r} \ldots x_{q}]$ which correspond to the processors having the same final destination $({x_{int}}, \lfloor y^{\prime} \rfloor)$ after $X - Y$ routing. The number of these subintervals is clearly $O(L)$ where $L$ is now given by (10) and computation time is also $O(L)$ for finding these intervals. Thus, eventually, the processor $(x_{int},y)$ has the same information as that it had when following the first implementation of $X$-routing.

It is also worth mentioning that the above two alternative implementations of $X$-routing actually lead to the same overall complexity for the RAR operation as will be clear after the analysis of the remaining steps of that operation.

#### *Y*-routing

At this step, the packets reach their final destinations, moving vertically, that is, in parallel with axis $Y$. After the end of $X$-routing, the packet that started from processor $(x,y)$ is at processor $(x_{int},y)$ where $x_{int}$ is the approximation of $x^{\prime}$ by the integer $\lfloor {x^{\prime}} \rfloor$ or $\lceil {x^{\prime}} \rceil$. As has been mentioned earlier, the proxy packets arriving at processor $(x_{int},y)$ are coming from the same block of the frame $I_{n}$ and this processor can estimate the parameters $a_{i1}, \ldots,a_{i8}$ of the bilinear transformation for the origin block. By finding $x$ from the first Eq. (4) and then replacing $x(= x_{int} + \delta)$ in the second equation, we finally get:

11 $$y^{\prime } = \frac{(a_{i6} a_{i3} - a_{i7} a_{i2} )y^{2} + (a_{i1} a_{i6} - a_{i2} a_{i5} + a_{i7} x_{int} - a_{i4} a_{i7} + a_{i3} a_{i8} )y + a_{i5} x_{int} - a_{i4} a_{i5} + a_{i1} a_{i8} }{a_{i3} y + a_{i1}}+ \frac{a_{i7} y + a_{i5} }{a_{i3} y + a_{i1} }\delta$$

where $\delta \in [- 1,1]$. Division by zero does not arise since the denominator $a_{i3} y + a_{i1}$ is always positive from Lemma 3.1.

Now, $Y$-routing is executed in two stages. In the first stage, the packet in the processor $(x^{\prime},y)$ is sent to the processor $(x^{\prime}, \lfloor {y^{\prime\prime}} \rfloor )$ where $y^{\prime\prime}$ is given by the following relation:

12 $$y^{\prime\prime} = \frac{{(a_{i6} a_{i3} - a_{i7} a_{i2})y^{2} + \;(a_{i1} a_{i6} - a_{i2} a_{i5} + a_{i7} x_{int} - a_{i4} a_{i7} +a_{i3} a_{i8})y + a_{i5} x_{int} - a_{i4} a_{i5} + a_{i1} a_{i8}}}{{a_{i3} y + a_{i1}}}$$

At the second stage, we take into account the term $\frac{{a_{i7} y + a_{i5}}}{{a_{i3} y + a_{i1}}}\delta$ as well as the truncation of coordinate $y^{\prime\prime}$ to the nearest smaller integer, i.e. $\lfloor {y^{\prime\prime}} \rfloor$. Notice also that all the packets residing in the processor $(x_{int},y)$ after $X$-routing have the same destination $(x_{int},y^{\prime \prime})$ during $Y$-routing and thus they can be easily combined into a single proxy packet again.

Next, we will describe the first stage of $Y$-routing.

**Figure 2:**
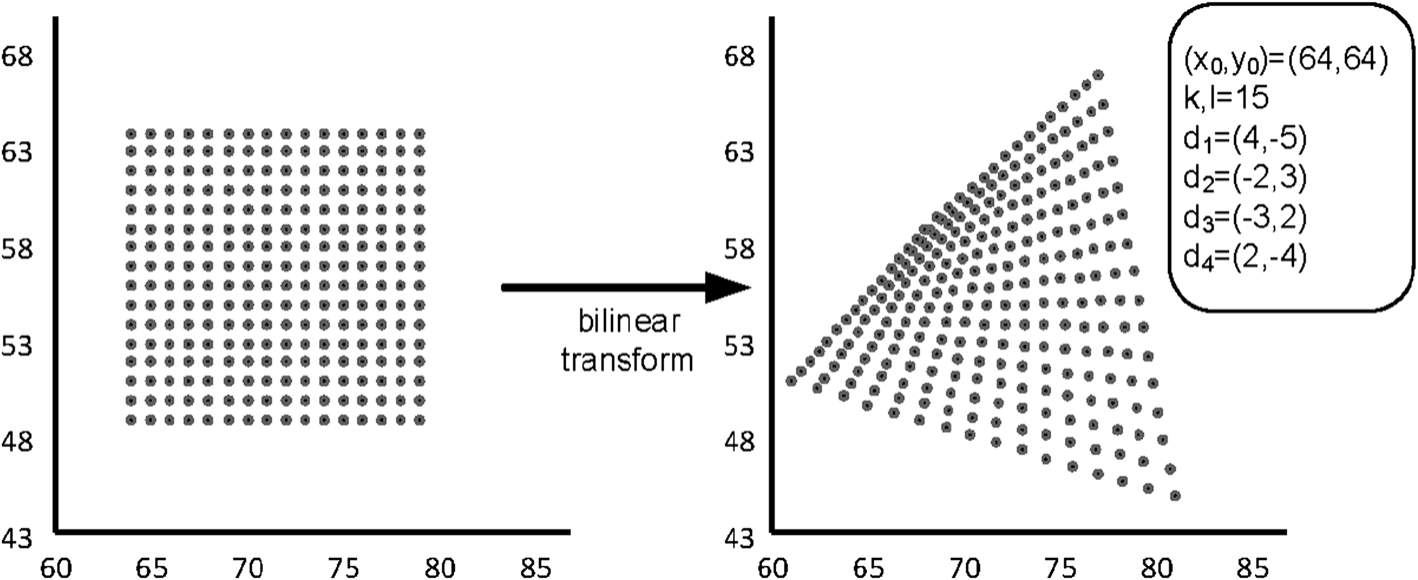
An example of the bilinear transformation on a frame block.

**Figure 3:**
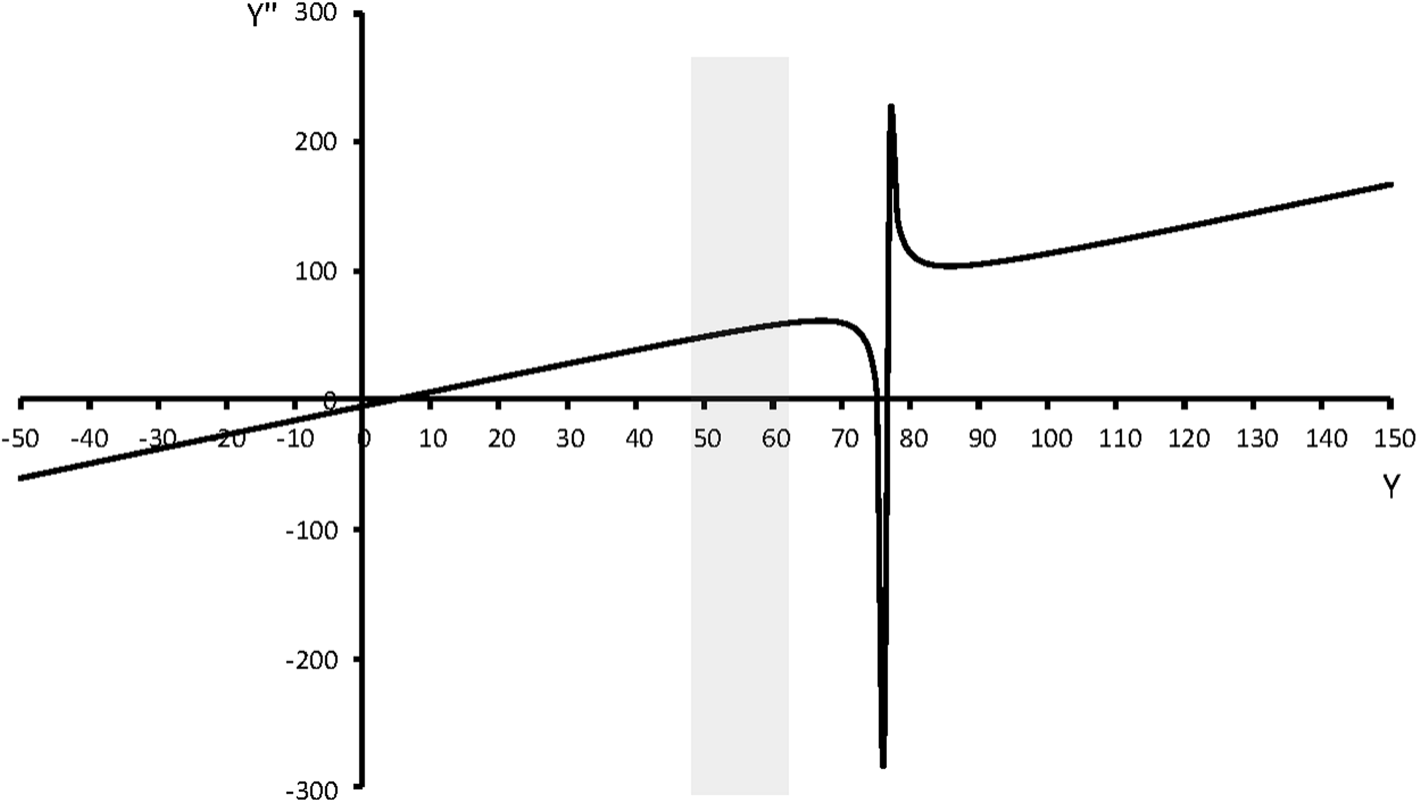
Graph of the function (12) for the example of Figure 2 and for $x_{int} = 72$.

*First stage*. The function (12) which gives the destinations of packets during this stage is a ratio of a second order polynomial over a linear function. Figure 2 depicts an example of the bilinear transformation on a block and Figure 3 illustrates the graph of $y^{\prime \prime}$ for this particular transformation. By following a standard analysis using the first derivative of this function and by taking into account that $y^{\prime \prime}$ is not continuous for $y$-values around the root of denominator, we can easily prove that the horizontal axis $y$ is always divided into at most four intervals where the function $y^{\prime \prime}$ is either increasing or decreasing. Let $(- \infty,y_{1}]$, $(y_{1},y_{2}]$, $(y_{2},y_{3}]$, $(y_{3}, + \infty)$ be these intervals. Obviously, $y_{1}$,$y_{2}$,$y_{3}$ can be easily determined by studying the first derivative of $y^{\prime \prime}$.

Here, it should be noted that actually we are not interested in the whole range of the values of $y$ but only for those $y$-values relevant for the corresponding block ($[y_{0} - l \ldots y_{0}]$), e.g., the shaded region in Figure 3. Although, it was not possible to prove it due to complexity of (12), however, by performing a number of tests with different parameters of the bilinear transformation for each test, we have noticed that within the relevant range $[y_{0} - l \ldots y_{0}]$, the function is monotone except for some cases where the block suffers severe distortion, e.g. when the left part of the block goes down and right part up and the two vertical sides nearly coincide. In that case, the function change monotonicity mode only once.

Thus, the general technique for the first stage of $Y$-routing is to split the packet routing into as many phases as the number of intervals with different monotonicity (at most four). At each phase, packets are sent only from those processor $(x^{\prime}_{int},y)$ whose $y$-coordinate belongs to the corresponding interval. Specifically, at the intervals where $y^{\prime \prime}$ is increasing, monotone routing is directly employed. At intervals where $y^{\prime \prime}$ is decreasing, each processor $(x^{\prime}_{int},y)$ first sends a packet to processor $(x^{\prime}_{int},N - 1 - y)$. This transfer can be easily done in $O(logN)$ time by complementing the bits of coordinate $y$. After this transfer, the packets to be sent are sorted in increasing order of their final destination $y^{\prime \prime}$ again. Thus, now monotone routing can be applied for packet routing.

In the discussion above, we implicitly assume that all packets are coming from the same initial block. However, the above techniques are still valid when there are packets from different blocks. For different initial blocks, the function (12) differs accordingly. Still, packet routing can be arranged in such a way that all packets to be sent in one of the at most four phases mentioned above will be sorted in increasing or decreasing order of their final destination again. This total ordering of packet destinations in each phase is thanks to the modification we did on $X$-routing step which ensures that each packet coming from a block will end up again in the same image block in the previous frame as well as because of the continuous motion model assumed in this work. According to that model, the blocks after the bilinear transformation maintain their initial relevant spatial placement. Specifically, we prove the following Lemma:

##### **Lemma 4.2**

*Let*$A$*and*$B$*be two packets from different initial blocks which are on the same column after the end of*$X$-*routing, specifically at processors*$({x_{int},y_{A}} )$*and*$({x_{int},y_{B}} )$*respectively. If*$y_{A} > y_{B}$*then*$y^{\prime \prime}_{A} > y^{\prime \prime}_{B}$*where*$y^{\prime \prime}_{A}$*and*$y^{\prime \prime}_{B}$*are given by* (*12*).

##### *Proof*

**Figure 4:**
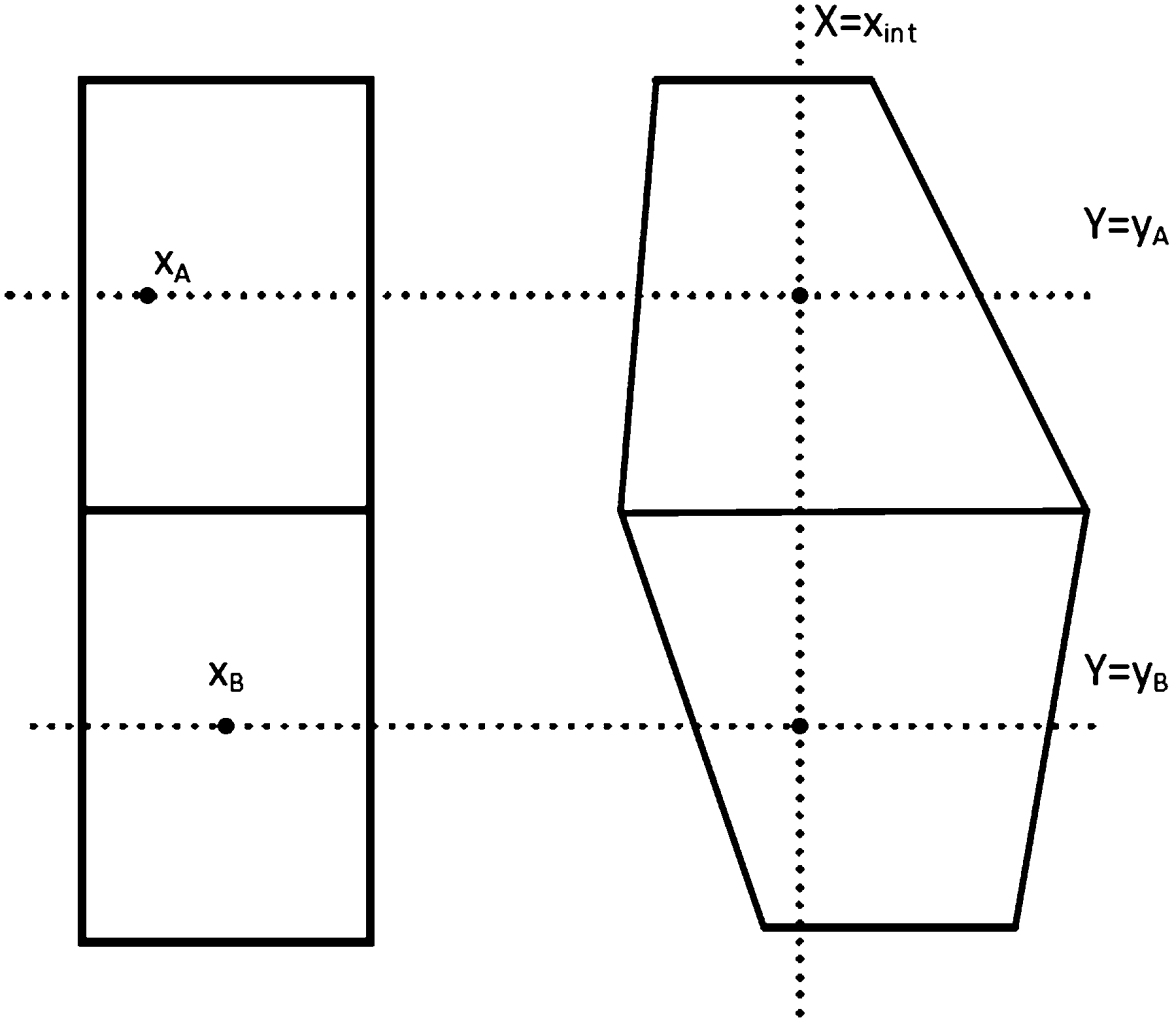
Proof of Lemma 4.2.

We will only consider the case where the packets $A$ and $B$ belong to adjacent initial blocks (Figure 4). Then, the general case is easily derived. Let $({x_{A},y_{A}} )$ and $({x_{B},y_{B}} )$ be two points inside the two blocks for which it holds that:

13 $$x_{int} = a_{A1} x_{A} + a_{A2} y_{A} + a_{A3} x_{A} y_{A} + a_{A4}$$

14 $$x_{int} = a_{B1} x_{B} + a_{B2} y_{B} + a_{B3} x_{B} y_{B} + a_{B4}$$

where $a_{A1}, \ldots,a_{A4}$ and $a_{B1}, \ldots,a_{B4}$ are the first four parameters of the bilinear transformation of the blocks of packets $A$ and $B$, respectively. The existence of these two points results from the properties of the bilinear transform and from the modification of $X$-routing which ensures that no packet will end up outside the image of its origin block after the application of the bilinear transformation. Note that $x$-coordinates of these points are not necessarily integer numbers.

Now, after applying the bilinear transformation, these points are mapped to the following points:

15 $$y^{\prime}_{A} = a_{A5} x_{A} + a_{A6} y_{A} + a_{A7} x_{A} y_{A} + a_{A8}$$

16 $$y^{\prime}_{B} = a_{B5} x_{B} + a_{B6} y_{B} + a_{B7} x_{B} y_{B} + a_{B8}$$

Due to continuous motion model, after the application of the bilinear transformation, the blocks maintain their initial relevant vertical order and hence it holds that $y^{\prime}_{A} > y^{\prime}_{B}$. After finding $x_{A}$ and $x_{B}$ from Eqs. (13, 14), respectively and then replacing these in the Eqs. (15, 16) we get:

$$y^{\prime}_{A} = \frac{(a_{A3} a_{A6} - a_{A2} a_{A7})y_{A}^{2} + (a_{A1} a_{A6} - a_{A2} a_{A5} + a_{A7} x_{int} - a_{A4} a_{A7} + a_{A3} a_{A8})y_{A} + a_{A5} x_{int} - a_{A4} a_{A5} + a_{A1} a_{A8}}{a_{A3} y_{A} + a_{A1}}$$

$$y^{\prime}_{B} = \frac{{{(a_{B3} a_{B6} - a_{B2} a_{B7})y_{B}^{2} +} { (a_{B1} a_{B6} - a_{B2} a_{B5} + a_{B7} x_{int} - a_{B4} a_{B7} +} a_{B3} a_{B8})y_{B} + a_{B5} x_{int} - a_{B4} a_{B5} + a_{B1} a_{B8}}}{{a_{B3} y_{B} + a_{B1}}}$$

Now, it is easy to see that $y^{\prime \prime}_{A} = y^{\prime}_{A}$, $y^{\prime \prime}_{B} = y^{\prime}_{B}$ and hence $y^{\prime \prime}_{A} > y^{\prime \prime}_{B}$. $\square$

From this Lemma, it is now clear that the monotone routing can now be applied for the first stage of $Y$-routing. Again, packet combining can be employed for replacing all packets heading for the same processor with a single proxy-packet. After the end of $X$-routing, all these packets have been stored in neighboring processors along the same column and thus, combining is easy to implement. As a result, the first step of $Y$-routing can run in $O(logN)$ time. If $(x,y_{A})$, $(x,y_{A} + 1)$, $(x,y_{A} + 2)$, $\ldots$, $(x,y_{B} - 1)$, $(x,y_{B})$ are the processors whose packets have the same destination during the first stage of $Y$-routing, then the proxy packet of all the packets residing in these processors should carry the maximum absolute value of the fraction $\frac{{a_{i7} y + a_{i5}}}{{a_{i3} y + a_{i1}}}\delta$ for $y \in [y_{A} \ldots y_{B}]$. This information which will be denoted by $y_{cr}$ will be used at the second stage of $Y$-routing. The factor $\delta$ is the truncation error during the $X$-routing and gets nearly random values in the interval $(- 1,\,1)$. It is also easy to see that $y_{cr} = O(L)$.

*Second stage*. After the first stage of $Y$-routing, the proxy of the read-request originated from the processor $(x,y)$ has ended up at the processor $(x_{int},\lfloor {y^{\prime\prime}} \rfloor )$. In the second stage of $Y$-routing, we take into account the term $\frac{{a_{i7} y + a_{i5}}}{{a_{i3} y + a_{i1}}}\delta$ in (11) as well as the truncation error due to the approximation of $y^{\prime \prime}$ with the integer $\lfloor{y^{\prime \prime}}\rfloor$.

Now, each processor which has received a proxy-packet uses the value of $y_{cr}$ stored in the proxy-packet for determining the pixels that should be gathered from the nearby processors. Specifically, processor $(x_{int},\lfloor {y^{\prime\prime}} \rfloor )$ needs to get pixels only from the processors $(x_{int}+r,\lfloor {y^{\prime\prime}} \rfloor+q )$ where $r = - 1,0,1$ and $q=-\lfloor {y_{cr}} \rfloor\ldots\lceil {y_{cr}}\rceil +1$. These pixels surely include all the pixels necessary for the estimation of interpolation function (3) for all packets whose proxy-packet ended up at processor $(x_{int},\lfloor {y^{\prime\prime}} \rfloor )$.

The above group of pixels can be transferred from the nearby processors to the processor $(x_{int},\lfloor {y^{\prime\prime}}\rfloor)$ by running $O(y_{cr})$ or, equivalently, $O(L)$ shift operations. The total time for this transfer is $O({LlogN})$ in the case of one-port capability. In the case of all-port capability, the shift operations can be pipelined and so the total time for the above transfer is reduced to $O({L + logN})$. Clearly, the local memory per processor required for storing the received pixels is $O(L)$.

We have concluded the description of the first phase of the RAR operation. Next, we present the second phase of this operation.

### The second phase of the RAR operation

This phase is essentially the reversal of the steps executed during the first phase. At the end of first phase of the RAR operation, each processor $O(x_{int},\lfloor {y^{\prime\prime}} \rfloor)$ has gathered $O(L)$ pixels that should be returned to the processors that asked for them. The second phase of the RAR operation starts by reversing the first stage of $Y$-routing and the size of packets transferred in this step is $O(L)$. Thus, the time required for this step is $O({LlogN})$ ($O({L + logN})$) at most in the case of the one-port (all-port) capability. After, this step, each processor stores $O(L)$ pixels at most in its local memory.

Next, the $X$-routing step is reversed. The processors have kept in their local memory the packets that received at the end of $X$-routing during the first phase of the RAR operation and now they are able to return to each processor $(x,y)$ only the pixels that this processor needs for estimating the interpolated value $I(x^{\prime},y^{\prime})$ where $x^{\prime}$,$y^{\prime}$ are given by the Eq. (4). As a result, the packets sent during this step, are all of size $O(1)$, while each processor $(x,y)$ should send packets to at most $O(L)$ processors horizontally. Therefore, the reverse $X$-routing requires $O({LlogN})$ ($O({L + logN})$) time in the case of one-port (all-port) capability.

Now, each processor $(x,y)$ has all the pixels it needs for estimating the interpolation function (3) and hence the intensity value of the pixel which the pixel $(x,y)$ is mapped to in the previous frame $\tilde{I}_{n - 1}$ with the application of the bilinear transformation.

Finally, we can prove the following Theorem:

#### **Theorem 4.1**

*The motion estimation based on the bilinear transformation between two successive video frames of dimension*$N \times N$*can be executed on a hypercube of*$N^{2}$*nodes in*$O({klLlogN})$*or*$O({kl(L + logN)})$*time at most assuming one*-*port or all*-*port capability respectively where*$L$*is given by* (*10*)*. The local memory required at each processor for this computation is*$O(L)$*at most. With the constraints (6) on the displacement vectors at the block corners, the above time and the space complexities become*$O(kl^{2} logN)$, $O(kl^{2} + kllogN)$*and*$O(l)$*respectively.*

#### *Proof*

The most costly operation in each of the $\varTheta (kl)$ iterations of the Algorithm 2 is the RAR operation whose time complexity is $O({LlogN})$ or $O({L + logN})$ for one-port or all-port capability, respectively while the local memory at each processor is $O(L)$ at most. Thus, the time and space complexities stated in the theorem easily follow. Recall also that $L = O(l)$ at most and this maximum arises only in the rather uncommon scenario where the corners of a block are almost collinear along a vertical line after applying the bilinear transformation. $\square$

With the one-port assumption, a nice feature of all communications used in the proposed algorithm such as, the prefix-sum, monotone routing or shift, is that they are normal algorithms (Leighton 1992), that is, at any step of these communications, only one hypercube dimension is used and successive dimensions are used in successive steps. Now, a well-known fact for the normal algorithms is that they can be simulated with the same asymptotic complexity in other hypercubic networks (butterfly, cube-connected-cycles, shuffle-exchange or de Bruijn network) of the same number of nodes (Leighton 1992). Thus, the proposed parallel motion estimation algorithm can be easily ported to other interconnection network models as well.

## Conclusions

We have presented a parallel algorithm for motion estimation for video coding based on the bilinear transformation. The algorithm runs on the the parallel model of the hypercube which has been widely used for parallel algorithm design in the literature. We have also provided complete analysis of the time and space complexity of the proposed algorithm. We have also shown that our algorithm can be used not only for the hypercube network but can also run on other hypercubic networks as well.

## Abbreviations

ASIC: application specific integration circuits
GOP: group of pictures
RAR: random access read
